# Neutrophil apoptosis: a marker of disease severity in sepsis and sepsis-induced acute respiratory distress syndrome

**DOI:** 10.1186/cc5090

**Published:** 2006-11-08

**Authors:** Léa Fialkow, Luciano Fochesatto Filho, Mary C Bozzetti, Adriana R Milani, Edison M Rodrigues Filho, Roberta M Ladniuk, Paula Pierozan, Rafaela M de Moura, João C Prolla, Eric Vachon, Gregory P Downey

**Affiliations:** 1 Department of Internal Medicine, Faculty of Medicine, Federal University of Rio Grande do Sul, Rua Ramiro Barcelos n° 2400, 4° andar, Porto Alegre, Rio Grande do Sul, 90035-003, Brazil; 2 Intensive Care Unit, Intensive Care Division, Hospital de Clínicas de Porto Alegre, Rua Ramiro Barcelos n° 2350, Porto Alegre, Rio Grande do Sul, 90035-903, Brazil; 3 Department of Social Medicine, Faculty of Medicine, Federal University of Rio Grande do Sul, Rua Ramiro Barcelos n° 2400, 4° andar, Porto Alegre, Rio Grande do Sul, 90035-003, Brazil; 4 Intensive Care Unit of Trauma and Neurosurgery, Hospital Cristo Redentor, Grupo Hospitalar Conceição, Rua Domingos Rubbo n° 20, Porto Alegre, Rio Grande do Sul, 91040-000, Brazil; 5 Intensive Care Unit, Hospital Dom Vicente Scherer, Complexo Hospitalar Santa Casa de Porto Alegre, Rua Annes Dias n° 285, Porto Alegre, Rio Grande do Sul, 90020-090, Brazil; 6 Faculty of Pharmacy, Federal University of Rio Grande do Sul, Avenida Ipiranga n° 2752, Porto Alegre, Rio Grande do Sul, 90035-003, Brazil; 7 Faculty of Pharmacy, Pontifícia Universidade Católica do Rio Grande do Sul, Avenida Ipiranga n° 6681 Prédio 12, Bloco A, sala 202, Porto Alegre, Rio Grande do Sul, 90619-900, Brazil; 8 Division of Respirology, Department of Medicine and Toronto General Hospital Research Institute of the University Health Network and University of Toronto, 11C-1183 NCSB, Toronto General Hospital, 585 University Avenue, Toronto, ON, M5G 2N2, Canada

## Abstract

**Introduction:**

Apoptosis of neutrophils (polymorphonuclear neutrophils [PMNs]) may limit inflammatory injury in sepsis and acute respiratory distress syndrome (ARDS), but the relationship between the severity of sepsis and extent of PMN apoptosis and the effect of superimposed ARDS is unknown. The objective of this study was to correlate neutrophil apoptosis with the severity of sepsis and sepsis-induced ARDS.

**Methods:**

A prospective cohort study was conducted in intensive care units of three tertiary hospitals in Porto Alegre, southern Brazil. Fifty-seven patients with sepsis (uncomplicated sepsis, septic shock, and sepsis-induced ARDS) and 64 controls were enrolled. Venous peripheral blood was collected from patients with sepsis within 24 hours of diagnosis. All surgical groups, including controls, had their blood drawn 24 hours after surgery. Control patients on mechanical ventilation had blood collected within 24 hours of initiation of mechanical ventilation. Healthy controls were blood donors. Neutrophils were isolated, and incubated *ex vivo*, and apoptosis was determined by light microscopy on cytospun preparations. The differences among groups were assessed by analysis of variance with Tukeys.

**Results:**

In medical patients, the mean percentage of neutrophil apoptosis (± standard error of the mean [SEM]) was lower in sepsis-induced ARDS (28% ± 3.3%; *n *= 9) when compared with uncomplicated sepsis (57% ± 3.2%; *n *= 8; *p *< 0.001), mechanical ventilation without infection, sepsis, or ARDS (53% ± 3.0%; *n *= 11; *p *< 0.001) and healthy controls (69% ± 1.1%; *n *= 33; *p *< 0.001) but did not differ from septic shock (38% ± 3.7%; *n *= 12; *p *= 0.13). In surgical patients with sepsis, the percentage of neutrophil apoptosis was lower for all groups when compared with surgical controls (52% ± 3.6%; *n *= 11; *p *< 0.001).

**Conclusion:**

In medical patients with sepsis, neutrophil apoptosis is inversely proportional to the severity of sepsis and thus may be a marker of the severity of sepsis in this population.

## Introduction

Sepsis is a leading cause of death in intensive care unit (ICU) patients [1], with an estimated incidence of 700,000 cases per year in the United States resulting in more than 200,000 deaths annually [2,3]. Acute respiratory distress syndrome (ARDS) is a frequent complication of sepsis [4-6]. The mortality rate of ARDS remains high, ranging between 20% and 60% [4,7-13]. Leucocytes, including neutrophils and macrophages, are believed to contribute to inflammatory tissue injury in sepsis and ARDS. It is hypothesised that unrestrained release of leucocyte-derived cytotoxic products contributes to injury of lungs and other organs [14-16]. A better understanding of the pathophysiology of sepsis and ARDS is essential for the treatment or prevention of these devastating conditions.

Apoptosis is involved in removal of senescent cells and is thought to be essential for the non-injurious resolution of inflammation [17-27]. The role of apoptosis in the pathophysiology of sepsis and multiple organ dysfunction syndrome (MODS) has been the focus of recent studies. There is evidence of an association between apoptosis and outcomes of patients with MODS [15,20,22,23,25,28]. Recent studies suggest that neutrophil apoptosis is decreased in systemic inflammatory response syndrome (SIRS) [28,29], sepsis [30-37], and ARDS [12,14,16,26,38-40]. The increased life span of neutrophils may be associated with increased tissue injury in these syndromes [12,14-16,20,22,29]. Currently, information on the relationship between neutrophil apoptosis and the severity of sepsis and sepsis-induced ARDS is incomplete [22,23,32-35,41]. Accordingly, the objective of the current study was to determine whether neutrophil apoptosis correlates with the severity of sepsis and sepsis-induced ARDS.

## Materials and methods

### Patient selection and study protocol

A prospective cohort study enrolled patients at three tertiary teaching hospitals in Porto Alegre city, southern Brazil, from January 2000 to December 2004. Patients were included in the study if they met criteria for sepsis and ARDS.

### Sepsis

Sepsis and its subsets were defined according to the Consensus Conference of the American College of Chest Physicians and the Society of Critical Care Medicine [1]. Sepsis, a systemic inflammatory response secondary to infection, was defined by two or more of the following criteria: (a) body temperature greater than 38°C or less than 36°C, (b) heart rate greater than 90 beats per minute, (c) respiratory rate greater than 20 breaths per minute or a PaCO_2 _(arterial partial pressure of carbon dioxide) less than 32 mm Hg, and (d) leucocytes greater than 12,000 cells per cubic millimetre, less than 4,000 cells per cubic millimetre, or greater than 10% bands. Septic shock was defined as sepsis-induced hypotension, despite adequate fluid resuscitation, along with the presence of hypoperfusion abnormalities or organ dysfunction. In our study, the term 'uncomplicated sepsis' was used for patients with sepsis according to the Consensus criteria instead of the more frequently used, but ambiguous, term 'sepsis.'

### ARDS

ARDS was defined according to criteria of the 1994 American-European Consensus Conference on ARDS [42]. These included acute hypoxemia, ratio of PaO_2 _(arterial partial pressure of oxygen) to FiO_2 _(fraction of inspired oxygen) of 200 mm Hg or less, bilateral infiltrates on chest x-ray, pulmonary artery wedge pressure less than or equal to 18 mm Hg, or no clinical evidence of left atrial hypertension.

### Control groups

1. Healthy controls were healthy blood donors (more than 18 years old) at the Hospital de Clínicas de Porto Alegre.

2. Surgical controls were patients submitted for elective surgery who had no evidence of infection, sepsis, or ARDS. Studies suggest that surgery itself has an influence on neutrophil apoptosis [43-46].

3. The mechanical ventilation (MV) group consisted of patients submitted to MV but without evidence of infection, sepsis, or ARDS. The objective was to verify whether the MV itself influenced neutrophil apoptosis. All patients of this group were on MV for a period of 24 hours.

### Exclusion criteria

Exclusion criteria were congestive heart failure, ARDS secondary to factors other than sepsis (for example, pancreatitis, burns, and multiple trauma), interstitial lung disease, use of immunosuppressive drugs (for example, corticosteroids), AIDS, malignancies, chronic inflammatory diseases (for example, rheumatoid arthritis), and transfusion of blood or blood products within the preceding 24 hours.

### Ethical issues

The study was approved by the hospitals' ethics committees, and informed consent was obtained from the patient or a surrogate and from the healthy volunteers.

### Sample and data collection

The venous blood sampling of medical patients was performed within 24 hours of diagnosis of sepsis and its subsets, ARDS, and for patients on MV. All surgical groups, including controls, had their blood drawn 24 hours after surgery. For healthy controls, a blood sample was obtained at the time of blood donation. The investigators followed each patient admitted to the ICU to identify patients who fulfilled the entry criteria. For each patient, a data record was completed and stored in a data bank.

### Study variables

#### Outcome variables

The primary outcome variable was mean percentage of neutrophil apoptosis.

#### Independent variables

Independent variables were age, gender, medical/surgical patient status, Acute Physiology and Chronic Health Disease Classification System II (APACHE II) score, total maximum sequential organ failure assessment (SOFA) score, organ system failure based on the SOFA score, and 28-day mortality from the time of entry into the study. If the patient was discharged from the hospital, mortality was assessed by telephone or mail.

### Study procedures

#### Neutrophil isolation

Human neutrophils (more than 98% pure) were isolated from whole blood using dextran sedimentation and discontinuous plasma-Percoll (Amersham Biosciences AB, now part of GE Healthcare, Little Chalfont, Buckinghamshire, UK) gradients as described previously [47]. The separation procedure required two hours, and the cells were used immediately after isolation for the experiments described. The functional integrity and non-activated state of isolated neutrophils have been validated in previous reports [47,48]. Neutrophil viability was greater than 97% using Trypan blue exclusion.

#### Neutrophil apoptosis

After isolation, neutrophils were washed twice and resuspended at a density of 1 × 10^6 ^cells per millilitre in RPMI 1640 with 10% foetal bovine serum, L-glutamine (2 mM), penicillin (100 mg/ml), and streptomycin (100 μg/ml) (Gibco, now part of Invitrogen Corporation, Carlsbad, CA, USA). Cells were then incubated at 37°C in a 5% CO_2 _atmosphere for 24 hours in polypropylene tubes to prevent adherence. Cell viability assessed by Trypan blue exclusion exceeded 97%. After 24 hours, neutrophils were sedimented by cytocentrifugation on a glass microscope slide as described below.

#### Quantification of neutrophil apoptosis

Neutrophil apoptosis was assessed by light microscopy (×200) analysis of cytospun cells stained with Wright's Giemsa method and identification of nuclear changes (condensation of chromatin and simplification of nuclear structure) characteristic of apoptosis [17,49,50]. Two blinded investigators assessed the percentage of neutrophil apoptosis on cytospun preparations by analysing 500 cells per slide each. The analysis was performed on two different slides from the same patient. Data were reported as the percentage of apoptotic cells. The percentage was obtained by using the mean value obtained by the two investigators.

To validate the light microscopic method of assessment of neutrophil apoptosis, we used a second independent method in healthy donors, annexin V binding with quantification by flow cytometry [51]. In brief, neutrophils (1 × 10^6^) were washed with ice-cold phosphate-buffered saline (PBS) and then incubated with fluorescein isothiocyanate (FITC)-conjugated annexin V (R&D Systems, Inc., Minneapolis, MN, USA) in the presence of propidium iodide (PI) for 30 minutes at 4°C. Cells were washed, resuspended in PBS, and analysed by flow cytometry (FACStar; Becton Dickinson, Mountain View, CA, USA). Cells that were FITC-positive and PI-negative were considered to be apoptotic. The extent of neutrophil apoptosis was compared with the percentage of neutrophil apoptosis determined by nuclear morphology and light microscopy (linear regression slope 0.87 R^2 ^= 0.968, *n *= 6). These results confirm the validity of Wright's Giemsa staining to assess apoptosis.

### Sample size

The sample size was calculated using data from the study patients because there was no information in the literature to help sample size estimation. The study power for the study comparisons was 90%.

### Data quality control

A database coordinator was responsible for monitoring all data collection and entry. All data were checked for any inconsistencies. A random sample of 20% of the records was selected and compared with the original data-collection forms to detect any data-entry errors.

### Statistical analysis

A stratified analysis was performed considering the status of medical or surgical patients. For each strata, the percentage of neutrophil apoptosis measured in the different groups was compared using one-way analysis of variance (ANOVA), considering that the study variables were normally distributed and that the variances were equal. All comparisons with a *p *value less than 0.05 were considered statistically significant. A *post hoc *Tukey test was used. Continuous variables, other than the percentage of neutrophil apoptosis, were also compared using ANOVA and the *post hoc *Tukey tests. For continuous variables comparing two groups, the Student *t *test was used. Categorical variables were compared using the χ^2 ^test. Correlation analysis (Pearson) was performed between the main outcome of neutrophil apoptosis and other continuous variables, including age and APACHE II and SOFA scores, stratified for medical and surgical status. All analyses were performed using the Statistical Package for Social Sciences, version 12 (SPSS Inc., Chicago, IL, USA).

## Results

A total of 57 patients and 64 controls were included in the study (see Table 1 for population characteristics). A detailed description of the diagnoses, sites of infection, microbiology, and sources of materials for culture from all patients is included in Table 2 (medical patients) and Table 3 (surgical patients).

The comparison of the percentage of neutrophil apoptosis was significantly different among all groups (*p *< 0.001; ANOVA). A stratified analysis was performed considering surgical/medical status. The mean percentage of neutrophil apoptosis (± standard error of the mean [SEM]) was significantly lower in the surgical controls (52% ± 3.6%) when compared with healthy controls (69% ± 1.1%; *p *= 0.001; Student *t *test).

In medical patients, a significant difference was observed in the age variable (Table 4). The control group was younger than the MV group (*p *= 0.02; Tukey test). A Pearson correlation test showed a weak and negative correlation (*p *= 0.35) between age and neutrophil apoptosis, suggesting that age did not have a major effect on the percentage of neutrophil apoptosis in this study (data not shown).

**Figure 1:**
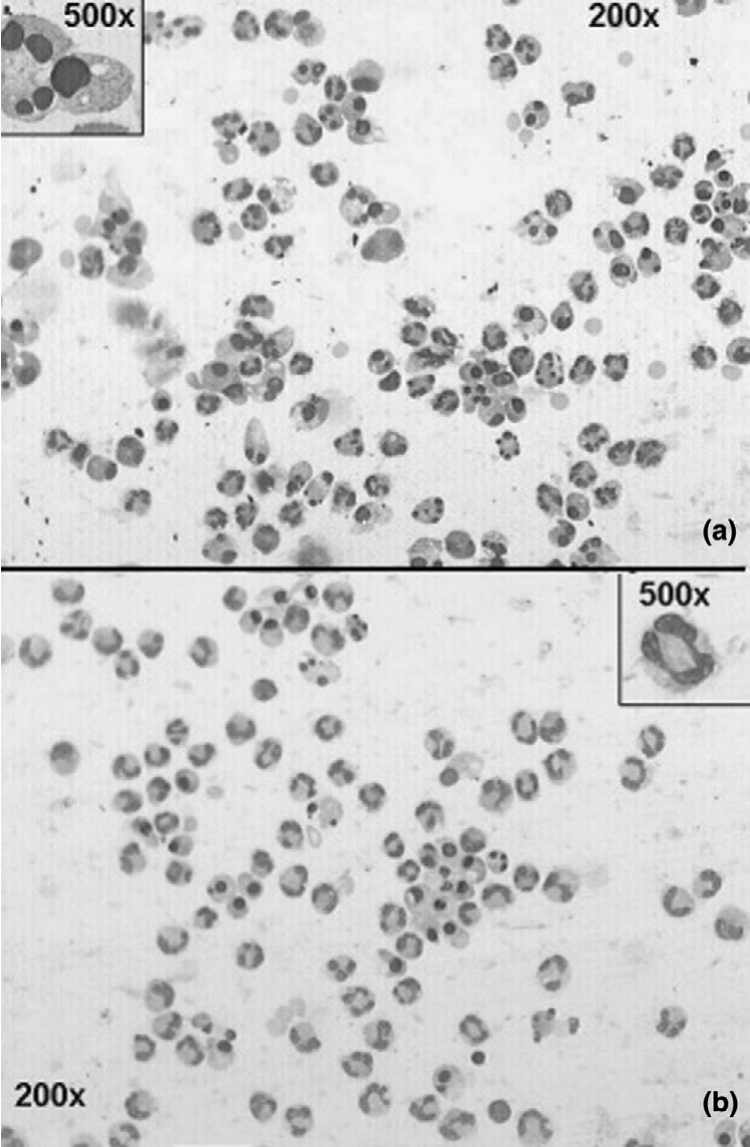
Apoptosis of neutrophils in a healthy donor and in a patient with sepsis-induced acute respiratory distress syndrome (ARDS). **(a) **Apoptosis of neutrophils in a healthy donor. Wright's Giemsa staining of cytocentrifuge smear shows predominance of cells in apoptosis. Inset shows morphological detail of an apoptotic cell, with loss of chromatin fine granularity (condensation) and karyorrhexis. **(b) **Apoptosis of neutrophils in a patient with sepsis-induced ARDS. Wright's Giemsa staining of cytocentrifuge smear shows predominance of normal-looking cells. Inset shows morphological detail of a normal cell, with fine granularity of chromatin and normal lobulated nucleus. Magnifications ×200 (insets ×500).

**Figure 2:**
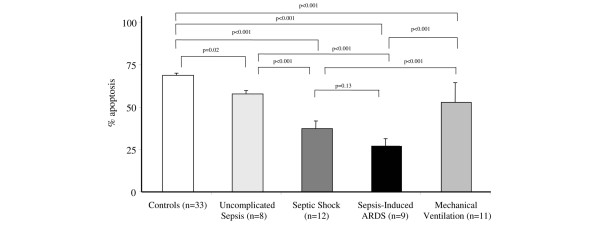
Mean percentage of neutrophil apoptosis in medical patients. There was a statistically significant difference among the groups (*p *< 0.001; analysis of variance). The differences between individual groups as determined by a *post hoc *Tukey test are illustrated. ARDS, acute respiratory distress syndrome.

Neutrophil apoptosis differed significantly among the groups of medical patients. Figure 1 shows images of neutrophil apoptosis in Wright's Giemsa-stained slides obtained from a healthy control (a) and from a patient with ARDS (b). The percentage of neutrophil apoptosis (± SEM) was lower in ARDS (28% ± 3.3%; *n *= 9) compared with uncomplicated sepsis (57% ± 3.2%; *n *= 8; *p *< 0.001), MV (53% ± 3.0%; *n *= 11; *p *< 0.001), and with healthy controls (69% ± 1.1%; *n *= 33; *p *< 0.001). However, it did not differ from septic shock (38% ± 3.7%; *n *= 12; *p *= 0.13) (Tukey test; Figure 2). In the septic shock group, the mean percentage of neutrophil apoptosis was significantly lower than in uncomplicated sepsis, MV, and healthy controls (*p *< 0.001; Tukey test). The mean percentage of neutrophil apoptosis was significantly lower in patients with uncomplicated sepsis (*p *= 0.02; Tukey test) and in the MV group (*p *< 0.001; Tukey test) compared with healthy controls. There was no difference in the mean percentage of neutrophil apoptosis between the uncomplicated sepsis and the MV groups (*p *= 0.8; Tukey test). These observations suggest that in medical patients, the severity of sepsis is inversely proportional to the mean percentage of neutrophil apoptosis (Figure 2).

Variables such as 28-day mortality and APACHE II and SOFA scores were also analysed in the medical groups (Table 4). Twenty-eight-day mortality was higher in the ARDS and septic shock groups when compared with the group with uncomplicated sepsis (Table 4). ARDS and septic shock groups had a higher mean SOFA score when compared with the other groups (*p *< 0.001; Tukey test) (Table 4). However, no statistical difference was observed between the ARDS and septic shock groups (*p *= 0.3; Tukey test).

Detailed data regarding number of organ dysfunctions/failures, according to SOFA score, are summarised in Table 4. Many patients with uncomplicated sepsis developed organ failure after blood sampling and during their hospitalisation in the ICU.

**Figure 3:**
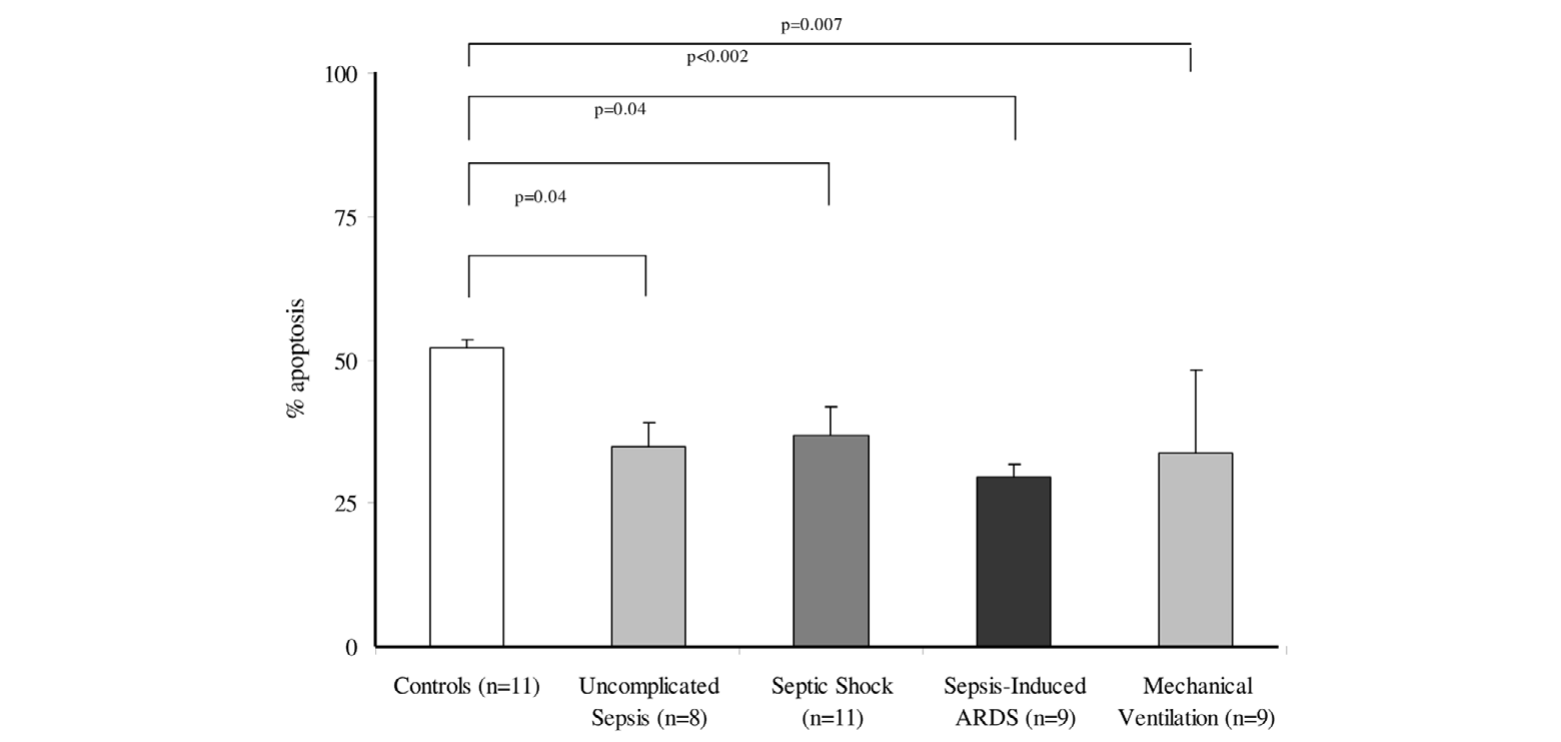
Mean percentage of neutrophil apoptosis in surgical patients. There were statistically significant differences among the groups (*p *< 0.001; analysis of variance). *Post hoc *Tukey test results are illustrated. ARDS, acute respiratory distress syndrome.

In surgical patients, the mean percentage of neutrophil apoptosis in all groups (uncomplicated sepsis [*p *= 0.04], septic shock [*p *= 0.04], ARDS [*p *< 0.002], and MV [*p *= 0.007] groups [Tukey test]) was significantly lower than in controls (Figure 3). No statistical difference was found among the mean percentage of neutrophil apoptosis of uncomplicated sepsis, septic shock, ARDS, and MV groups. Other variables were also analysed in surgical groups (Table 5).

We attempted to perform a subgroup analysis based on the different degrees of severity of sepsis in medical and surgical patients to ascertain whether there was an association between neutrophil apoptosis and mortality. This was not successful, probably due to the small sample size studied. A moderate and negative correlation between the mean SOFA score and the percentage of neutrophil apoptosis in medical patients was observed (R = -0.56; *p *< 0.001), indicating that the lower the mean percentage of apoptosis, the higher the mean SOFA score. However, in surgical patients, this correlation was weak and not statistically significant.

## Discussion

The primary observation of the current study is that the extent of neutrophil apoptosis correlates inversely with the severity of sepsis and sepsis-induced ARDS in medical patients. Neutrophils from medical patients with uncomplicated sepsis, septic shock, and ARDS displayed lower degrees of apoptosis as compared with controls. Furthermore, we observed a progressive decrease in neutrophil apoptosis as the severity of sepsis increased. This is the first study to correlate the extent of apoptosis of peripheral blood neutrophils with the severity of sepsis and ARDS.

Our study confirms and extends the previous reports of decreased neutrophil apoptosis in patients with sepsis and ARDS with or without sepsis [30-33,35,36,38-40]. One study reported that neutrophil apoptosis was decreased in patients with sepsis compared with healthy controls [33]. However, that study combined patients with different degrees of severity of sepsis into one large group (labelled 'sepsis') that was compared with healthy controls but did not correlate the extent of neutrophil apoptosis with the severity of sepsis. Other studies that examined apoptosis of circulating neutrophils from septic patients assessed only one level of severity of sepsis (for example, only severe sepsis [30-32] or MODS [35]). Another study examined the rates of apoptosis of neutrophils in bronchoalveolar lavage fluid (BALF) of septic patients and demonstrated decreased apoptosis when all cells (including neutrophils) from the BALF were analysed *ex vivo *[36].

In patients with ARDS, our study is in agreement with previous studies that have demonstrated decreased neutrophil apoptosis in patients with ARDS, including those with sepsis-induced ARDS [38-40]. Several studies have documented that BALF recovered from patients during the early stages of both septic and non-septic ARDS is able to prolong the life span of neutrophils incubated *ex vivo *and that this effect may be ascribable to elevated levels of cytokines such as granulocyte-colony stimulating factor, granulocyte macrophage-colony stimulating factor (GM-CSF), and interleukin (IL)-2 [38-40]. Interestingly, Matute-Bello and colleagues [39] reported that higher GM-CSF levels in BALF correlated with survival in patients with ARDS. The authors suggested that this effect may not be related to modulation of neutrophil apoptosis but rather due to effects on other cells such as alveolar macrophages and epithelial cells. Lesur and colleagues [40] also demonstrated that exposure of normal blood neutrophils to BALF from patients with ARDS delayed apoptosis *in vitro*. In general, these results are in agreement with our observations and indicate that modulation of apoptosis of neutrophils and other lung cells is an early phenomenon in the inflammatory milieu of the lung in sepsis. It is noteworthy that our study is the first to evaluate apoptosis of peripheral blood neutrophils specifically from patients with sepsis-induced ARDS.

The mechanisms responsible for the decreased neutrophil apoptosis in sepsis and ARDS are incompletely understood. One potential mechanism involves activation of nuclear factor-κB with a concomitant reduction of the activity of caspases 3 and 9, and maintenance of mitochondrial transmembrane potential [33]. Other possible mechanisms involve modulation of Mcl-1 (myeloid cell leukaemia-1) [32], PBEF (pre-B cell colony-enhancing factor) [35], and p38 mitogen-activated protein kinase (p38 MAPK) [41] signalling pathways.

The current study was stratified (medical/surgical status) because previous studies have suggested that surgery *per se *may influence neutrophil apoptosis [43-46]. Additionally, because MV has been shown to affect apoptosis in other cell types [52-57], we included a control group of patients (medical and surgical) submitted to MV but who had no history of infection, sepsis, or ARDS.

We observed that the extent of neutrophil apoptosis was significantly lower in the surgical controls when compared with medical controls, an effect that has been reported by others [43-45]. Indeed, we observed a decrease in neutrophil apoptosis in all surgical groups. However, there was no statistical difference between these groups. Therefore, the correlation between neutrophil apoptosis and the severity of sepsis observed in medical patients was not observed in the surgical groups. There are several factors that might account for the decreased neutrophil apoptosis in surgical patients, including effects of anaesthesia and of the localised tissue trauma related to the surgical procedure with release of cytokines such as IL-6 [43] and IL-8 [45]. In this regard, a recent study [58] examined the effects of surgery on Fas-induced neutrophil apoptosis and reported that the anti-apoptotic action of plasma was not affected by the addition of neutralising antibodies to GM-CSF, IL-6, or IL-8, indicating that these cytokines are not a dominant factor mediating the anti-apoptotic effects on Fas-induced apoptosis in surgical patients. However, the anti-apoptotic effect of plasma was attenuated by pharmacological inhibitors of either PI3 kinase or extracellular signal-regulated kinase (ERK), but not by a p38 MAPK inhibitor, implicating PI3 kinase and ERK in the signalling pathway mediating the anti-apoptotic effect of plasma under the conditions described above. Another study demonstrated a decrease in apoptosis of exudative neutrophils obtained from peritoneal fluid from patients with recent gastrointestinal surgery [44]. In contrast, a recent report describes enhanced apoptosis of peripheral blood neutrophils of patients undergoing elective surgery under general anaesthesia [46]. Taken together, alterations in neutrophil function which occur in the post-operative period may predispose to untoward outcomes via modulation of the complex inflammatory response to surgery.

Previous studies support the concept that injurious modes of MV *per se *may result in release of inflammatory mediators that lead to inflammatory lung injury [52,53,59,60]. In support of this notion, we observed that neutrophil apoptosis was diminished in the group of patients subject to MV but without evidence of infection, sepsis, or ARDS. However, our results also indicate that MV *per se *did not account for the low percentage of neutrophil apoptosis observed in the group of patients with more complicated sepsis. The effect of MV extends beyond the lungs to other organs and has been termed 'biotrauma' [54,55]. Imai and collaborators [52] documented effects of MV on epithelial cell apoptosis in the lung as well as in the kidneys and small intestine, the former accompanied by biochemical evidence of organ dysfunction. A previous study from our group demonstrated that BALF obtained from ARDS patients ventilated with injurious MV activated neutrophil oxidant production and release of elastase, effects that correlated to the degree of lung injury and systemic inflammatory response and to multiple organ failure [61]. Although the effect of BALF on neutrophil apoptosis was not assessed in this study, we predict that it would decrease apoptosis. The 'biotrauma' hypothesis is supported by evidence from experimental models, including humans [59], animals [62], isolated lung [54], and stressed cell systems [63].

We observed that the mortality rate was higher in medical patients with ARDS, followed by septic shock, when compared with the uncomplicated sepsis group. To understand the significance of these mortality rates, we used instruments such as the total maximum SOFA score to quantify the severity of illness. From a correlation test evaluating the association between the mean percentage of neutrophil apoptosis and the mean SOFA score, two correlations merit further consideration: (a) the correlation between the severity of sepsis and the percentage of neutrophil apoptosis and (b) the association among the severity of sepsis, percentage of neutrophil apoptosis, and mortality. The correlation analysis suggests an inverse association between disease severity and the percentage of neutrophil apoptosis. Because the mean SOFA score correlates with mortality [64,65], our findings suggest that there is an association between the severity of sepsis, the extent of neutrophil apoptosis, and mortality.

We did not observe an association between neutrophil apoptosis and mortality in the current study. One limitation in this regard is that the sample size was not sufficient to assess such an association. However, the observed results of the mean percentage of neutrophil apoptosis, the mean SOFA score, and the mortality rates suggest that the higher the mortality rate (and disease severity), the lower the percentage of neutrophil apoptosis. In future studies with a larger sample size, it will be important to evaluate whether the percentage of neutrophil apoptosis is associated with mortality within the different degrees of severity of sepsis.

### Study limitations

The decrease in the percentage of neutrophil apoptosis may not be specific for sepsis and ARDS. In fact, it appears that any event resulting in SIRS (such as sepsis) has the potential to affect the immune system, including neutrophil survival and function. However, the patients included in our study, including the controls groups, were carefully selected to allow us to study the specific correlation between neutrophil apoptosis and sepsis. Our results demonstrate that in medical patients with sepsis, neutrophil apoptosis is inversely proportional to the severity of sepsis. The correlations of neutrophil apoptosis with other causes of SIRS, if any, require further study.

The observations in the current study represent an important first step to a better understanding of the influence of sepsis on neutrophil apoptosis by defining the clinical associations of differing degrees of neutrophil apoptosis in this milieu. However, the observational design of the current study did not allow us to explore the possible mechanism(s) such as the role of specific receptors and intracellular signalling pathways in modulation of neutrophil apoptosis during sepsis. Further studies will be important to address these issues and will provide important information on the signal transduction pathways modulating neutrophil apoptosis during sepsis.

## Conclusion

We observed that in medical patients with sepsis, neutrophil apoptosis is inversely proportional to the severity of this syndrome, including ARDS. In surgical patients, the mean percentage of neutrophil apoptosis for all sepsis groups was significantly lower than in the control group, but was not proportional to the severity of sepsis. These observations suggest that in medical patients, neutrophil apoptosis may be a marker of the severity of sepsis. We speculate that an influx of long-lived neutrophils may contribute to enhanced inflammatory injury to the lungs and other organs. The identification of specific mechanisms of neutrophil apoptosis in sepsis, including sepsis-induced ARDS, may lead to new strategies to improve the survival of those patients and patients with other inflammatory disorders in which neutrophils have been directly implicated.

## Key messages

• In medical patients with sepsis, neutrophil apoptosis is inversely proportional to the severity of sepsis.

• In surgical patients with sepsis, the rate of apoptosis was lower than in controls but was not proportional to the severity of sepsis.

• These observations suggest that in medical patients, neutrophil apoptosis may be a marker of the severity of sepsis.

## Abbreviations

ANOVA = analysis of variance; APACHE II = Acute Physiology and Chronic Health Disease Classification System II; ARDS = acute respiratory distress syndrome; BALF = bronchoalveolar lavage fluid; ERK = extracellular signal-regulated kinase; FITC = fluorescein isothiocyanate; GM-CSF = granulocyte macrophage-colony stimulating factor; ICU = intensive care unit; IL = interleukin; MODS = multiple organ dysfunction syndrome; MV = mechanical ventilation; p38 MAPK = p38 mitogen-activated protein kinase; PBS = phosphate-buffered saline; PI = propidium iodide; SEM = standard error of the mean; SIRS = systemic inflammatory response syndrome; SOFA = sequential organ failure assessment.

## Competing interests

The authors declare that they have no competing interests.

## Authors' contributions

LF participated in the design, coordination, data collection, and analysis of the study and helped to draft the manuscript. LFF performed the study and helped to draft the manuscript. MCB participated in the study design, performed the statistical analysis, and helped to draft the manuscript. ARM, EMRF, RML, PP, RMM, and EV participated in the acquisition of the data for the study. JCP helped in neutrophil apoptosis assessment and digital imaging of Wright's Giemsa slides. GPD participated in the study design and development and helped to draft the manuscript. All authors read and approved the final manuscript.

## Figures and Tables

**Table 1:** Characteristics of the study population according to group allocation

Variables	Uncomplicated sepsis (*n *= 16)	Septic shock (*n *= 23)	Sepsis-induced ARDS (*n *= 18)	Mechanical ventilation (*n *= 20)	Controls (*n *= 44)	*P *value^a^
Age (years, mean ± SEM)	57 ± 3.3	57 ± 4.5	46 ± 4.4	54 ± 3.5	43 ± 1.8	0.002
Male/Female (percentage)	62.5/37.5	52.2/47.8	50/50	55/45	50/50	0.93
Medical/Surgical (percentage)	50/50	52.2/47.8	50/50	55/45	75/25	-

^a^Analysis of variance or χ^2 ^test. ARDS, acute respiratory distress syndrome; SEM, standard error of the mean.

**Table 2:** Detailed description of the medical patients

Patient	Group			
Uncomplicated sepsis (*n *= 8)				

	Diagnosis	Site of infection	Microorganism	Material

1	Pneumonia/COPD	Respiratory	Not identified	Sputum/Blood
2	Pneumonia/COPD	Respiratory	*Staphylococcus aureus*	Blood	
3	Pneumonia/Stroke	Respiratory	Not identified	Sputum/Blood
4	Pneumonia/Guillain-Barre syndrome	Respiratory	*Enterobacter sp*	Sputum
5	Pneumonia/Subarachnoid hemorrhage	Respiratory	*Staphylococcus sp*	Blood
6	Pneumonia/DM/Pickwick syndrome	Respiratory	Not identified	Sputum/Blood
7	Pneumonia/Head trauma	Respiratory	*Pseudomonas aeruginosa*	Sputum
8	Pneumonia/Intracerebral hemorrhage	Respiratory	*S. aureus*	Sputum
				

Septic shock (*n *= 12)				

	Diagnosis	Site of infection	Microorganism	Material

1	Pneumonia/COPD	Respiratory	*S. aureus*	Blood
2	Pneumonia	Respiratory	*S. aureus*	Sputum
3	Pneumonia/COPD	Respiratory	*S. aureus*	Blood
4	Pneumonia/COPD	Respiratory	*P. aeruginosa/Haemophilus influenzae*	Sputum
5	Pneumonia/UTI	Respiratory/Urinary	Not identified/*Klebsiella pneumoniae*	Sputum/Urine
6	Pneumonia/UTI/DM	Respiratory/Urinary	Not identified/*Candida sp*	Sputum/Blood and urine
7	UTI/SBP/Cirrhosis	Abdominal/Urinary	*S. aureus *and *Streptococcus viridans/Enterococcus faecium, S. viridans*, and *Escherichia coli*	Ascites/Urine
8	Pneumonia	Respiratory	Not identified	Sputum/Blood
9	Pneumonia/COPD	Respiratory	Not identified	Sputum/Blood
10	Meningitis	CNS	*Neisseria meningitis*	Liquor/Blood
11	UTI/Lyell syndrome	Urinary/Skin	*Enterococcus sp/Acinetobacter sp*	Urine/Skin secretion
12	Pneumonia	Respiratory	*S. aureus*	Blood
				

Sepsis-induced ARDS (*n *= 9)				

	Diagnosis	Site of infection	Microorganism	Material

1	Pneumonia/Leptospirosis	Respiratory	*Enterobacter sp*	Sputum
2	Pneumonia/Suicide attempt (glicosate ingestion)	Respiratory	*P. aeruginosa*	Sputum
3	Pneumonia/UTI/Diarrhea	Respiratory, Urinary, and Intestinal	Not identified/*K. pneumoniae/E. coli OH157*	Sputum/Urine/Feces
4	Pneumonia/UTI/DM	Respiratory/Urinary	*Streptococcus agalactiae, S. aureus/Staphylococcus sp, E. coli*	Blood/Urine
5	Pneumonia	Respiratory	*Haemophilus sp*	Sputum
6	Septic arthritis	Joint	*S. aureus*	Sinovial liquid
7	Pneumonia	Respiratory	Not identified	Sputum/Blood	
8	Pneumonia	Respiratory	*E. coli, Moraxella sp*	Sputum
9	Pneumonia/COPD	Respiratory	Not identified	Sputum/Blood
				

Mechanical ventilation (*n *= 11)				

	Diagnosis			

1	Anaphylaxis (anaesthesia)			
2	Head trauma			
3	Spinal cord trauma			
4	Subarachnoid hemorrhage			
5	Subarachnoid hemorrhage			
6	Intracerebral hemorrhage			
7	Anaphylaxis (anaesthesia)			
8	Guillain-Barre syndrome			
9	Intracerebral hemorrhage			
10	Anaphylaxis (anaesthesia)			
11	Epilepsy			

ARDS, acute respiratory distress syndrome; CNS, central nervous system; COPD, chronic obstructive respiratory disease; DM, diabetes mellitus; SBP, spontaneous bacterial peritonitis; UTI, urinary tract infection.

**Table 3:** Detailed description of the surgical patients

Patient	Group				
Uncomplicated sepsis (*n *= 8)					

	Diagnosis	Site of infection	Microorganism	Material	Surgery

1	Pneumonia/Intracerebral haemorrhage	Respiratory	*Pseudomonas aeruginosa*	Sputum	Craniotomy
2	Pneumonia/COPD	Respiratory	*P. aeruginosa*	Sputum	Aortic-femoral bypass
3	Pneumonia/Perforated ulcer	Abdominal	*Candida albicans*	Ascites	Laparatomy
4	Cholangitis/UTI	Abdominal/Urinary	*Enterococcus sp/Escherichia coli*	Blood/Urine	Exploratory laparotomy
5	Pneumonia/Peritonitis/Colonic perforation due to colonoscopy	Respiratory/Abdominal	*Enterobacter sp*/Not identified	Sputum/Blood	Exploratory laparotomy
6	Cholecystitis	Abdominal	*Enterococcus sp*	Blood	Cholecystectomy
7	Pneumonia/Intracerebral haemorrhage	Respiratory	*Enterobacter sp*, *Haemophilus sp*, and *Staphylococcus aureus*	Sputum	Craniotomy
8	Pneumonia/Stroke/UTI/Celulitis	Respiratory/Urinary/Skin	Not identified/*Enterobacter sp/S. aureus*	Sputum/Urine/Skin secretion	Abdominal aortic aneurysm repair
					

Septic shock (*n *= 11)					

	Diagnosis	Site of infection	Microorganism	Material	Surgery

1	Pneumonia	Respiratory/Catheter	*P. aeruginosa/S. aureus*	Sputum/Catheter	Carotid aneurysm repair
2	Diverticulitis/UTI	Abdominal/Urinary	Not identified/*Candida sp*	Blood/Urine	Small bowel resection with anastomosis
3	Perforated peptic ulcer/Cirrhosis/Alcohol abuse	Abdominal	*S. aureus*	Blood/Ascites	Laparatomy
4	Septic arthritis (Hip)	Joint	*Streptococcus agalactiae*	Joint fluid	Surgical drainage
5	Pneumonia/Head trauma (subdural haematoma)	Respiratory	Not identified	Sputum/Blood	Craniotomy
6	Pyelonephritis/Nephrolitiasis/Neurogenic bladder	Urinary	*Staphylococcus sp/P. aeruginosa*	Blood/Urine	Nephrectomy and abscess drainage
7	Perforated peptic ulcer	Abdominal	*S. aureus*, *Streptococcus viridans*, and *Enterobacter sp*	Ascites	Exploratory laparotomy
8	Pneumonia/Endocarditis/Intracerebral haemorrhage	Respiratory/Heart	*Pseudomonas sp*/Not identified	Sputum/Blood	Craniotomy
9	Pneumonia/COPD/UTI Oesophageal laceration	Respiratory/Urinary	*S. aureus *and *Acinetobacter sp*/*E. coli*	Sputum/Urine	Oesophageal laceration repair
10	Cholangitis	Abdominal	*E. coli*	Blood	Exploratory laparotomy
11	Peritonitis/Perforated peptic ulcer	Abdominal	*Enterococcus sp*, *Candida sp*, and *S. aureus*	Ascites	Laparotomy
					

Sepsis-induced ARDS (*n *= 9)					

	Diagnosis	Site of infection	Microorganism	Material	Surgery

1	Cholangitis	Abdominal	*Klebsiella pneumoniae*	Ascites	Hepatic artery aneurysm ligation
2	Diverticulitis	Abdominal	*E. coli*	Blood	Small bowel resection with anastomosis
3	Pneumonia	Respiratory	*P. aeruginosa*	Sputum	Pleurostomy closure
4	Pneumonia	Respiratory	*Staphylococcus coag neg*	Blood	C-section
5	Septic arthritis	Hip joint	*Staphylococcus haemolyticus*	Blood	Hip drainage
6	UTI/Intestinal fistula	Urinary	*Candida sp *and *Enterococcus sp*	Blood and urine	Intestinal fistula closure
7	Pneumonia/UTI/Peripheral vascular disease	Respiratory/Urinary/Skin	Not identified/*Candida sp/S. aureus*	Sputum/Urine/Skin secretion	Above-knee amputation
8	Septic arthritis	Hip joint	*Stenotroptomonas maltophilia *and *Staphylococcus coag neg/S. agalactiae*	Blood and joint fluid	Hip drainage
9	Cholecystitis	Abdominal/Catheter	*E. coli/S. aureus*	Blood/Catheter	Cholecystectomy
					

Mechanical ventilation (*n *= 9)					

	Diagnosis	Surgery			

1	Head trauma (subdural haematoma)	Craniotomy			
2	Intracerebral haemorrhage	Craniotomy			
3	Head trauma (subdural haematoma)	Craniotomy			
4	Head trauma (subdural haematoma)	Craniotomy			
5	Head trauma (epidural haematoma)	Craniotomy			
6	Uterine leyomioma	Hysterectomy			
7	Abdominal trauma	Exploratory laparotomy			
8	Head trauma (subdural haematoma)	Craniotomy			
9	Intracerebral haemorrhage	Craniotomy			
					

Controls (*n *= 11)					

	Surgery				

1	Humeral prostheses				
2	Inguinal hernia repair				
3	Septoplasty				
4	Inguinal hernia repair				
5	Arthrodesis (tibia-tarsus)				
6	Tibial osteosynthesis				
7	Septoplasty				
8	Diaphragmatic hernia repair and laparoscopic fundoplication				
9	Iliofemoral bypass				
10	Incisional hernia repair				
11	Septoplasty				

ARDS, acute respiratory distress syndrome; COPD, chronic obstructive respiratory disease; UTI, urinary tract infection.

**Table 4:** Characteristics of the medical patients

	Uncomplicated sepsis (*n *= 8)	Septic shock (*n *= 12)	Sepsis-induced ARDS (*n *= 9)	Mechanical ventilation (*n *= 11)	Controls (*n *= 33)	*P *value
Age (years, mean ± SEM)	50.8 ± 4.9	56 ± 5.6	43.2 ± 5.8	57.5 ± 4.9	37.1 ± 1.7	<0.008^a^
Male/Female (percentage)	50.0/50.0	58.3/41.7	44.4/55.6	36.4/63.6	48.5/51.5	0.7^b^
APACHE II score (percentage)	13.6 ± 2.2	21 ± 2.2	21.5 ± 1.2	-	-	0.1
Maximum SOFA score (percentage)	4.7 ± 0.7	9.9 ± 1.0	12 ± 0.8	-	-	<0.001^a^
Organ dysfunction (percentage)						
0	50	25	0	-	-	-
1	0	16.7	77.8	-	-	-
2	25	41.7	0	-	-	-
3	25	16.7	22.2	-	-	-
Organ failure (percentage)						
0	25	0	0	-	-	-
1	62.5	25	0	-	-	-
2	12.5	41.7	55.6	-	-	-
3	0	33.3	33.3	-	-	-
4	0	0	11.1	-	-	-
Mortality in 28 days (percentage)	37.5	75	77.8	-	-	-
Neutrophil apoptosis (mean percentage ± SEM)	57 ± 3.2	38 ± 3.7	28 ± 3.3	53 ± 3.0	69 ± 1.1	<0.001^a^

^a^*P *value from the comparisons using analysis-of-variance test; ^b^*p *value from the comparisons using χ^2 ^test. APACHE II, Acute Physiology and Chronic Health Disease Classification System II; ARDS, acute respiratory distress syndrome; SEM, standard error of the mean; SOFA, sequential organ failure assessment.

**Table 5:** Characteristics of the surgical patients

	Uncomplicated sepsis (*n *= 8)	Septic shock (*n *= 11)	Sepsis-induced ARDS (*n *= 9)	Mechanical ventilation (*n *= 9)	Controls (*n *= 11)	*P *value
Age (years, mean ± SEM)	64.0 ± 3.3	58 ± 7.4	49.5 ± 6.9	51 ± 5	45.9 ± 5.5	0.1
Male/Female (percentage)	75/25	45.5/54.5	55.6/44.4	77.8/22.2	54.5/45.5	0.5^a^
APACHE II score (percentage)	15.3 ± 1.8	21.1 ± 1.9	21.7 ± 3.9	-	-	0.1
Maximum SOFA score (percentage)	6.8 ± 1.1	10 ± 1.3	12.2 ± 0.8	-	-	<0.001^b^
Organ dysfunction (percentage)						
0	0	9.1	11.1	-	-	-
1	50	18.2	44.4	-	-	-
2	37.5	36.4	22.2	-	-	-
3	12.5	18.2	22.2	-	-	-
4	0	18.2	0			
Organ failure (percentage)						
0	12.5	9.1	0	-	-	-
1	62.5	36.4	11.1	-	-	-
2	25	36.4	66.7	-	-	-
3	0	18.2	22.2	-	-	-
Mortality in 28 days (percentage)	62.5	45.5	66.7	-	-	-
Neutrophil apoptosis (mean percentage ± SEM)	35 ± 3.2	36 ± 5.2	29 ± 2.1	32 ± 3.9	52 ± 3.6	<0.001^b^

^a^*P *value from the comparisons using χ^2 ^test; ^b^*p *value from the comparisons using analysis-of-variance test. APACHE II, Acute Physiology and Chronic Health Disease Classification System II; ARDS, acute respiratory distress syndrome; SEM, standard error of the mean; SOFA, sequential organ failure assessment.
